# Physical Fitness, Body Composition, and Adherence to the Mediterranean Diet in Young Football Players: Influence of the 20 mSRT Score and Maturational Stage

**DOI:** 10.3390/ijerph17093257

**Published:** 2020-05-07

**Authors:** Samuel Manzano-Carrasco, Jose Luis Felipe, Javier Sanchez-Sanchez, Antonio Hernandez-Martin, Leonor Gallardo, Jorge Garcia-Unanue

**Affiliations:** 1 Faculty of Sport Sciences, University of Castilla-La Mancha, 45004 Toledo, Spain; samuel.manzano@uclm.es (S.M.-C.); antoniohmds@gmail.com (A.H.-M.); Leonor.Gallardo@uclm.es (L.G.); Jorge.GarciaUnanue@uclm.es (J.G.-U.); 2 School of Sport Sciences, Universidad Europea de Madrid, 28670 Villaviciosa de Odon, Spain; javier.sanchez2@universidadeuropea.es

**Keywords:** physical activity, soccer, health promotion, sport, children, adolescents, nutrition

## Abstract

This study aimed to analyze the differences in physical fitness variables, body composition, and adherence to the Mediterranean diet according to the cardiorespiratory fitness and the maturational stage in young football players. A total of 194 male football players (aged 8–16) from three football sport schools participated in this study. Data on cardiorespiratory fitness (the 20-m shuttle run test), anthropometric measurements, handgrip strength, respiratory capacity (forced spirometry), and adherence to the Mediterranean diet (KIDMED questionnaire) were collected. Players were divided into two groups depending on their maturational stage (prepubertal *n* = 127 and pubertal *n* = 67). The results show a direct relationship between low levels of cardiorespiratory fitness and body mass index, as well as body fat and leg fat. Similarly, players with lower cardiorespiratory fitness presented higher values of handgrip strength in the prepubertal state. On the other hand, improvements in respiratory values were observed in the pubertal state with the rest of the parameters when the cardiorespiratory fitness was increased. Therefore, the promotion of recreational football that encourage and develop cardiorespiratory fitness is a key factor and can be used as an effective sport activity to promote physical fitness and healthy habits in children and adolescents as well as within the population that is already physically active.

## 1. Introduction

Scientific evidence has shown that physical and sports activity practiced regularly at a moderate–vigorous intensity is one of the best strategies currently available to promote the welfare of the population and public health [1]. This practice has a positive relationship with having an optimal quality of life in childhood, adolescence, and adulthood [2]. In addition, levels of a sedentary lifestyle and childhood obesity are reduced, which are worldwide public health problems [3] that lead to a higher risk of suffering from a cardiovascular and metabolic disease in adulthood [4]. Thus, early age is one of the most important periods of life, since the health status is influenced by several factors, including healthy eating habits and physical activity along with a large number of physiological and psychological changes [2].

In the context of the promotion of healthy habits through sport, football (soccer) is probably one of the most demanded disciplines worldwide, with more than 400 million active federate players around the world [5]. However, it is important to distinguish between competitive football in which there is competition where performance is required, and recreational football, which can be defined as the practice of free-form football, without a competitive aim, but rather for recreational, formative, and health purposes. Currently, recreational football is possibly one of the most widely practiced sports, particularly in demand by the school-aged population and adolescents.

The implications of recreational football are emerging interest because physical activity and physical exercises are essential for healthy growth and development. Moreover, recreational football has a great potential to improve the health and physical fitness levels [6]. This may derive from the dynamic nature of football which not only provides opportunities to develop different aspects of physical fitness of its participants, but it also contains positive motivational and social factors that contribute to the maintenance of a physically active lifestyle [7]. Some studies have evaluated the effect of recreational football on different health variables in children and adolescents [8,9,10]. For example, Vicente-Rodriguez et al. [8] revealed that football participation is beneficial at a prepuberal age as players showed to have less body fat and better physical fitness than their sedentary counterparts. In this way, a study by Krustrup et al. [11] showed that the practice of recreational football in short periods improves the musculo-skeletal, metabolic, and cardiovascular systems. Thus, the benefits intrinsically associated with regular practice of recreational football could vary depending on different age categories and could be improved if the main basic physical abilities such as cardiorespiratory fitness [12], or cardiovascular or muscular adaptations [13] are controlled. However, although there are many studies on the conditioning factors and characteristics of health-related physical fitness in children and youth [14], these kind of studies on patterns in the active population and specific sports such as football are less frequent [15]. New studies are necessary to analyze health-oriented physical fitness and healthy habits in this kind of population in order to obtain information for the development of specific policies in sport schools.

One of the best ways to distinguish the beneficial effect of playing football is physical fitness which is understood as the ability of an individual to perform physical activity and exercise [14] and is a powerful marker of health [16]. Indeed, physical fitness is an integrated and effective measure of body structures and functions such as musculoskeletal, cardiorespiratory, hemato-circulatory, endocrine-metabolic, and psycho-neurological which are involved in the proper performance of human activities [17]. Between these functions one of the main exponents of physical fitness is cardiorespiratory fitness [18] defined as the ability of an individual to perform long-term physical activity, or more specifically, a phenotype influenced by the interaction between intensity and volume with which physical efforts are performed. To determine cardiorespiratory fitness, one of the most used tests in different sports is the test know as Course Navette or the 20-m shuttle run test (20 mSRT) [19]. Some studies that used this test in young people have shown the benefits of cardiorespiratory fitness being a powerful physiological indicator of health, especially cardiovascular, metabolic, and respiratory functions [16]. Furthermore, higher levels of cardiorespiratory fitness have been associated with a healthier cardiovascular profile later in life, and this cardiovascular level along with muscle strength and body composition during childhood and adolescence is associated with a healthier cardiovascular profile and a lower risk of death later in life [20]. In this way, it is important to know the different levels of cardiorespiratory fitness to improve the performance of the players as well as their health, taking into account how a good weight status and healthy eating habits are important factors of present and future health [20]. Thus, it highlights the importance of factors such as a body composition, which can be manipulated through adherence to the Mediterranean diet, and it is a mainly indicating the constant and variable rates of fat-free mass, bone mass, and body fat in the body [21]. The Mediterranean diet has been considered a model of healthy diet [22], demonstrating a reduction in cardiovascular mortality in subjects adhering to this eating pattern [23]. In its original definition, the traditional Mediterranean diet (MD) features a high consumption of unrefined fruits, vegetables, legumes, and cereals, moderate-high consumption of olive oil and fish, moderate consumption of dairy products, and low consumption of meats [24]. Therefore, due to the importance of the weight status, eating habits, as well as an appropriate level of physical fitness for preventing diseases and shaping the health-related quality of life in an active young population, the aim of this study was to analyze the differences in physical fitness variables, body composition, and adherence to the Mediterranean diet according to the cardiorespiratory fitness and the maturational stage in young football players.

## 2. Materials and Methods

### 2.1. Participants

A total of 194 male participants enrolled in three municipal football sport schools in the province of Toledo (region in the center of Spain) and successfully completed the study (age range: 8–16 years old; age average: 12 ± 2). The study was conducted during the academic period when participants attended extracurricular football training sessions two times per week. For participation in the study, we included all players whose parents/legal guardians authorized by informed consent the inclusion of their children or guardians. Coaches, parents, and players were previously informed about the purpose of the study and the nature of the tests that would be performed through an informative document. Players were evaluated individually on a single occasion during a day of training. All participants trained 2 days a week for at least two hours. Indeed, maturity is necessary for studies on the growth of children [25]. Thus, all participants were divided into two groups depending on their pubertal status. A Marshall and Tanner test [26] was conducted for all participants. For this purpose, the sample was divided in prepubertal (*n* = 127) and pubertal (*n* = 67). This tool has been frequently used for the classification in different maturational stage in young and adolescent population [27]. It consists of 5 stages of pubic hair and testicular development in boys. According to these criteria, 2 groups were formed: prepubertal (Tanner I) and pubertal (Tanner II, III, and IV). The Tanner pubertal status was determined by self-assessment. This method correlated with a physician assessment of pubertal development. The researchers have been previously formed and gained experience in the use of this tool in previous studies. Furthermore, years and hours per week of planned out-of-school physical activity were registered.

The study was conducted in adherence to the standards of the Declaration of Helsinki (2013 review, Brazil) following the European Community’s guidelines for Good Clinical Practice (111/3976/88 of July 1990), as well as the Spanish legal framework for clinical research on humans (Real Decretory 561/1993 on clinical trials). The informed consent and the study were approved by the Bioethics Committee for Clinical Research of the Virgen de la Salud Hospital of Toledo and by the supervisors of the University of Castilla-La Mancha (REF:508/14072020).

### 2.2. Measure

*a*  *Anthropometric Parameters and Body Composition*
  An anthropometric assessment was carried out. A portable segmental analyzer of multifrequency body composition (Tanita MC-780, Tanita Corp., Tokyo, Japan) was used to measure weight (kg), body fat (%), and skeletal muscle mass (%). Furthermore, legs fat (%) and legs skeletal muscle mass (%) based on total weight for each leg were included. The body mass index (BMI) was calculated with the weight (kg) divided by the squared height of the participants. Height (cm) was measured with a stadiometer (Seca 214, Hamburg, Germany). Players were assessed with clothes and without shoes.
*b*  *Physical Fitness*
  The different parameters of physical fitness were assessed following the protocols of the ALPHA health-related fitness battery [17]. All the participants were familiar with all physical performance tests as they had previously developed familiarization sessions in physical education classes.

First, cardiorespiratory fitness was evaluated by the 20 mSRT. This maximum and progressive test measures the maximum cardiorespiratory fitness [28]. Players were required to run, in a straight line, between two lines distanced 20 m apart, while keeping a pace with the acoustic signals from a speakerphone audio player with Bluetooth technology. According to the protocol by Léger et al. [29], the initial speed was 8.5 km·h^−1^, which was increased by 0.5 km·h^−1^ each min (1 min = one stage). The test was finished when the athletes failed to reach the end lines before the audio signal on two occasions or when stopped because of fatigue. Players were allowed to perform the test once. The total time in min was retained. The results are presented both in min (stage) and percentile adjusted by sex and age, based in the Eurofit reference values for Castilla-La Mancha [30]. This test was performed at the end of the evaluation sessions, so that fatigue did not interfere with the other tests. The results of this test were used to divide the sample into two subgroups in relation to the level of cardiorespiratory fitness. For this, all participants with a result equal to or greater than the 75th percentile (≥P_75_) for their age were classified as having a good level of cardiorespiratory fitness and the rest as having a low level (<P_75_) [31].

Respiratory capacity was recorded through forced spirometry which is a physiological test that measures how a subject inhales or exhales volumes of air as a function of time [32]. Each of the players performed a maximum inspiration using a spirometer, no more than two seconds of apnea, and a maximum expiration until there is no air left in the lungs. The most important aspects of spirometry are the forced vital capacity (FVC), which is the volume delivered during an expiration made as forcefully and completely as possible starting from full inspiration, and the forced expiratory volume (FEV) in one second, which is the volume delivered in the first second of an FVC maneuver [33]. Other spirometric variables were also assessed and included in the analysis. Two measurements were implemented for each participant and the best measurement was preserved.

To evaluate the upper-body muscular strength, the handgrip strength test (kg) was applied using a dynamometer with an adjustable grip (TKK 5001 Grip A; Tokyo, Japan). Players had to continuously tighten the grip for 2 s with the elbow position in full extension. The test was repeated twice (right hand and left hand, alternately). The best score of the two attempts for each player was chosen to the nearest 1 g [17]. The results are presented both in kg and percentile adjusted by sex and age, based on the Eurofit reference values for Castilla-La Mancha [30].

*c*  *Adherence to the Mediterranean Diet*
  In order to determine the adherence to the Mediterranean diet and the existence of possible eating disorders, the KIDMED questionnaire was used. This test, previously validated, consists of 16 items where twelve items represent a positive score for the adherence to the Mediterranean diet and the remaining 4 items represent a negative score [33]. A positive answer to a question that involves greater adherence to the diet is worth +1 point. A positive answer to a question that means less adherence to the diet is worth –1 point. Negative answers do not score (a value of 0 is noted). The KIDMED index is the sum of all the scores and ranges from 0 to 12 points (minimum to maximum adherence to the Mediterranean diet). The adherence to the Mediterranean diet could be categorized as low adherence (very low-quality diet, 0–3), medium adherence (improvement of the diet is needed, 4–7), and high adherence (ideal adherence to the Mediterranean diet, 8–12) [33]. However, in this study, the total score (KIDMED index) was used as scale variable [34].

### 2.3. Statistical Analysis

Data are presented as means ± standard deviations. All data were statistically analyzed using SPSS V24.0 for Windows (SPSS Inc., Chicago, IL, USA). The level of significance was set at *p* < 0.05. Before carrying out the analyses, the Kolmogorov–Smirnov distribution test was performed to confirm a normal distribution of the variables. Differences between groups were evaluated through two-way ANOVA (prepubertal vs. pubertal and <P_75_ cardiorespiratory fitness vs. ≥P_75_ cardiorespiratory fitness). All anthropometric measurements, respiratory variables, handgrip strength, and adherence to the Mediterranean diet were used as dependent variables. Post-hoc analysis was adjusted by the Bonferroni method. Furthermore, Effect sizes (Cohen’s d, ES) were calculated and defined as follows: trivial, <0.19; small, 0.2–0.49; medium, 0.5–0.79; large, >0.8 [35].

## 3. Results

Table 1 shows the descriptive data of the participants (anthropometric variables, body composition, respiratory capacity, cardiorespiratory fitness, handgrip strength, and KIDMED index).

The results showed significant differences between the maturational stage in all variables (*p* < 0.05), except FEV_1_/FVC, handgrip percentile and KIDMED index, and significant differences between cardiorespiratory fitness level in weight, BMI, total body fat, total muscle mass, as well as fat mass and muscle mass in both legs (*p* < 0.05). However, interaction effects between the combinations of both factors were found in all variables (*p* < 0.05), except FEV_1_/FVC and KIDMED index. Therefore, post-hoc pairwise comparisons are presented in Table 2 and Table 3.

Table 2 presents the results in relation to body composition variables. Prepubertal players showed significant lower weight, height, and BMI than pubertal players in both cardiorespiratory fitness levels (*p* < 0.05; ES from 0.41 to 1.92). Furthermore, significant differences between cardiorespiratory fitness levels were found in weight, height, and BMI in the prepubertal group (*p* < 0.05; ES from 0.44 to 1.07); however, only differences in BMI were found in the pubertal group (*p* = 0.018; IC: 1.74 to 3.81; ES: 0.57).

Prepubertal players showed a significantly higher percentage of total body fat (24.50%) compared to pubertal players (21.93%) in the <P_75_ cardiorespiratory fitness group (*p* = 0.018; Confidence Interval (CI): 0.45 to 4.71; Effect Size (ES): 0.42). Furthermore, a significantly lower percentage of total muscle mass was registered in the prepubertal group (71.45%) compared to the pubertal group (74.03%), as well as in the <P_75_ cardiorespiratory fitness group (*p* = 0.012; CI: −4.51 to −0.57; ES: 0.45). However, no significant differences in total body fat or total muscle mass were found between the maturational stage groups in the ≥P_75_ cardiorespiratory fitness level (*p* > 0.05). On the other hand, players with <P_75_ cardiorespiratory fitness revealed a higher percentage of total body fat in the prepubertal group (24.50%) and pubertal group (21.39%) compared to players with ≥P_75_ cardiorespiratory fitness (pubertal: 19.87%; prepubertal: 17.88%) with medium to large effect sizes (*p* < 0.001; CI: 2.74 to 6.53; ES: 0.90 for the prepubertal group and *p* = 0.004; IC: 1.32 to 6.78; ES: 0.77 for the pubertal group). Opposite differences were found for total muscle mass, where the <P_75_ cardiorespiratory fitness group presented significantly lower values than ≥P_75_ both in prepubertal and pubertal groups (*p* < 0.01; ES: 0.77 to 0.88).

Regarding the data obtained from the lower extremities of the players, the prepubertal group revealed a higher percentage of fat mass and a lower percentage of muscle mass in both legs and in both cardiorespiratory fitness levels (*p* < 0.05; ES: 0.54 to 0.93). The <P_75_ cardiorespiratory fitness group showed a significantly higher percentage of fat mass and a lower percentage of muscle mass than the ≥P_75_ cardiorespiratory fitness group in pubertal participants (*p* < 0.05; ES: 0.47 to 0.67). However, in prepubertal participants only, significant differences were found in the fat mass percentage (*p* < 0.05; ES: 0.76 to 0.79).

Table 3 presents the results in relation to respiratory variables, handgrip strength, and adherence to the Mediterranean diet. Prepubertal players showed significantly lower values in all respiratory variables except FEV_1_/FVC (*p* < 0.05; ES: 0.44 to 2.12). FVC presented higher values in the <P_75_ cardiorespiratory fitness group (2.82 l) than in the ≥P_75_ cardiorespiratory fitness group (2.48 l) in prepubertal players (*p* = 0.005; IC: 0.11 to 0.58; ES: 0.57). Conversely, FVC and FEV_1_ showed lower values in the <P_75_ cardiorespiratory fitness group (FVC: 3.66 l and FEV_1_: 3.14 l) than in the ≥P_75_ cardiorespiratory fitness group (FVC: 4.02 l and FEV_1_: 3.56 l) in pubertal players (*p* = 0.044; IC: −0.70 to −0.01; ES: 0.43 for FVC and *p* = 0.006; IC: −0.73 to 1.13; ES: 0.63 for FV_1_).

The prepubertal group evidenced significantly lower values of handgrip strength than the pubertal group in all variables, except handgrip strength (percentile) in the ≥P_75_ cardiorespiratory fitness group (*p* < 0.01; ES: 0.69 to 1.78). Handgrip strength (kg) presented higher values in the <P_75_ cardiorespiratory fitness group (23.21 kg) than in the ≥P_75_ cardiorespiratory fitness group (19.80 kg) in prepubertal players (*p* = 0.013; IC: 0.72 to 6.09; ES: 0.49).

No significant differences were found in the KIDMED index, although effect sizes close to medium are found, with a higher index in the ≥P_75_ cardiorespiratory fitness group both in pubertal participants (ES: 0.41).

Finally, Table 4 shows the results of the influence of different variables on cardiorespiratory fitness. In addition to age, percentage of muscle mass and FVC have a positive and significant relationship with stages of 20 mSRT, while BMI has a negative and significant relationship (*p* < 0.05). Handgrip strength (kg) and the KIDMED index show a positive reaction, although it is not significant (*p* > 0.05).

## 4. Discussion

The results of this study suggested that there are differences in anthropometric, respiratory, physical fitness variables, and adherence to the Mediterranean diet depending on the level of cardiorespiratory fitness in young football players. Furthermore, these differences vary slightly depending on the maturational stage. The scientific literature has already shown that cardiorespiratory fitness varies greatly with development, age, maturity, and gender and is highly related to both body size and composition [36].

In recent years, it has been shown that body composition is one of the most important indicators of a player’s physical state. Therefore, fat free mass, bone mass, and body fat are the most important variables [21]. The results of the study show that the groups with lower levels of cardiorespiratory fitness presented higher values in BMI and body fat. The scientific literature has already shown the importance of physical activity in reducing childhood obesity [25,37]. Moreover, Ubago et al. [38] declared that playing football reduces body fat levels and BMI in youth development, while Mota et al. [39] showed an increase in lean mass and leg strength after a six-month football program. It is important to highlight that important health problems in adults have been detected along with high BMI and percentage of body fat values during prepubertal and pubertal ages [40]. This entails a consequent economic cost imposed on the health system [41]. Therefore, improving health and physical fitness in this population should become the main strategy to reduce the risk of future mortality, looking for a healthy body weight [42]. Thus, promoting recreational football practice during development could be a very powerful strategy to combat obesity and its immediate and future problems [43].

An improvement in respiratory values was detected once the pubertal state was reached. In the same direction, certain improvements were related in the rest of the parameters (FVC; PEF; FEV_1_; FEV_1__FVC; FEF_25–75_) in this age group when the cardiorespiratory fitness was increased. Age, weight, height, and sex are some factors that affect lung capacity [44]. This explains the differences in favor of the pubertal group versus the prepubertal group. Specifically, the results show that football players who are in the pubertal stage have better FVC values than players in the prepubertal stage. This is undoubtedly due to greater progress and better cardiorespiratory capacity in this population thanks to its development and its practice of exercise [45]. In contrast, prepubertal stage players who have a low cardiorespiratory fitness obtained significantly higher values for FVC. This may occur due to the high anthropometric values of the age group (especially weight and body fat), positively affecting cardiorespiratory capacity. Therefore, it is important to determine the factors that affect respiratory functions and its control to achieve a good state of health during these stages of development [46]. Furthermore, this may also be due to the differences found in height in the prepubertal group, coinciding that the low cardiorespiratory fitness group presented higher height, which could affect FVC. The practice of sport is a key factor to increase capacity and motivation towards exercise to improve peak expiratory flow as well as quality of life and healthy habits [47].

Regarding adherence eating habits, this study revealed good adherence to the Mediterranean diet in the prepubertal and pubertal groups, as is the case in similar studies [23,33]. These results showed a clear tendency to maintain or increase patterns related to this type of diet since the different groups showed moderate adherence to the Mediterranean diet. Thus, a healthy an active lifestyle based on regular exercise is associated with better nutritional habits and a higher score in the KIDMED questionnaire [48]. Finally, higher values of handgrip strength in players with lower cardiorespiratory fitness were detected in the prepubertal group, although there were no significant differences in handgrip strength in the different cardiorespiratory fitness groups. This fact could be explained that this prepubertal group showed higher anthropometric values, and therefore, a higher BMI, or even a greater volume of body fat, may positively affect manual strength at an early age [49].

This study presents several limitations: (i) the anthropometry test with a portable segmental analyzer of multifrequency body composition, used in field and not laboratory research; (ii) it is important to highlight that the socioeconomic status can affect the acquisition of good healthy habits and adherence to the Mediterranean diet. Despite these limitations, the main strength of our study is that, to our knowledge, it is one of the few studies showing the influence of cardiorespiratory fitness with other variables related to health that are improved in young people who play recreational football. These data from young football players aged 8–6 years complement the evidence that suggests the importance of the maturational stage and practicing of daily physical activity or sport, such as recreational football, as well as maintaining good active and healthy lifestyle habits.

## 5. Conclusions

This study shows that children and adolescents who play recreational football have different anthropometric and respiratory values as well as handgrip strength depending on their cardiorespiratory fitness. Furthermore, these differences are higher in the pubertal stage than in the prepubertal stage. Taken together, these results suggest that the practice of physical and sports activity is an important habit that must be acquired in the prepubertal age in order to consolidate skills in the pubertal age. Moreover, promotion of sport exercises that encourage cardiorespiratory fitness is a key factor and can be used as an effective activity to promote physical fitness and healthy habits in children and adolescents, as well as within the population that is already physically active.

## Figures and Tables

**Table 1:** Characteristics of the participants.

Variables	Total	Prepubertal	Pubertal
Age (years)	11.96 ± 1.94	10.80 ± 1.18	14.15 ± 0.93
Weight (kg)	44.45 ± 13.00	39.06 ± 9.94	54.60 ± 12.02
Height (cm)	149.90 ± 12.67	144.04 ± 10.12	160.93 ± 9.16
Total BMI (kg/m^2^)	19.41 ± 3.34	18.59 ± 2.92	20.95 ± 3.57
Total body fat (%)	21.43 ± 5.79	21.94 ± 5.59	20.49 ± 6.07
Total muscle mass (%)	74.36 ± 5.46	73.81 ± 5.24	75.39 ± 5.74
Fat mass in left leg (%)	4.51 ± 1.14	4.68 ± 1.07	4.20 ± 1.22
Muscle mass in left leg (%)	12.34 ± 1.22	12.05 ± 1.11	12.88 ± 1.24
Fat mass in right leg (%)	4.56 ± 1.15	4.75 ± 1.08	4.20 ± 1.21
Muscle mass in right leg (%)	12.83 ± 1.29	12.50 ± 1.15	13.44 ± 1.33
FVC (L)	3.03 ± 0.89	2.63 ± 0.62	3.79 ± 0.81
PEF (L/s)	4.73 ± 1.34	4.41 ± 1.32	5.31 ± 1.19
FEV_1_ (L)	2.64 ± 0.76	2.30 ± 0.56	3.29 ± 0.68
FEV_1_/FVC (%)	85.94 ± 8.67	85.80 ± 9.99	86.19 ± 5.44
FEF_25–75_ (L/s)	3.06 ± 1.01	2.69 ± 0.86	3.75 ± 0.90
20 mSRT (stages)	6.53 ± 1.95	5.90 ± 1.69	7.73 ± 1.85
20 mSRT (percentile)	69.83 ± 20.29	72.75 ± 20.19	64.30 ± 19.46
Handgrip strength (kg)	25.06 ± 9.22	21.38 ± 7.12	31.99 ± 8.77
Handgrip strength (percentile)	61.37 ± 29.76	65.15 ± 29.42	54.26 ± 29.31
KIDMED index	7.14 ± 1.95	7.14 ± 2.01	7.13 ± 1.84

Abbreviations: BMI—body mass index; FVC—forced vital capacity; PEF—peak expiratory flow; FEV_1_—forced expiratory volume; FEF_25–75_—mean forced expiratory flow between 25% and 75% of FVC; L—liters; L/s,—liters per second.

**Table 2:** Comparisons of body composition between pubertal status and cardiorespiratory fitness.

	Cardiorespiratory Fitness		Effect Size		
	<P_75_	≥P_75_	<P_75_ vs. ≥P_75_	Prepub vs. Pub. <P_75_	Prepub vs. Pub. ≥P_75_
**Prepubertal**					
Weight (kg)	43.78 ± 10.89 *^,†^	35.28 ± 7.36 ^†^	0.93	1.02	1.92
Height (cm)	146.46 ± 10.30 *^,†^	142.09 ± 9.68 ^†^	0.44	1.53	1.87
Total BMI (kg/m^2^)	20.13 ± 3.03 *^,†^	17.35 ± 2.17 ^†^	1.07	0.41	1.06
Total body fat (%)	24.50 ± 5.51 *^,†^	19.87 ± 4.81	0.90	0.42	0.46
Total muscle mass (%)	71.45 ± 5.17 *^,†^	75.72 ± 4.53	0.88	0.45	0.52
Fat mass in left leg (%)	5.12 ± 1.12 *^,†^	4.32 ± 0.89 ^†^	0.79	0.54	0.66
Muscle mass in left leg (%)	11.95 ± 0.97 ^†^	12.12 ± 1.21 ^†^	0.16	0.67	0.93
Fat mass in right leg (%)	5.18 ± 1.11 *^,†^	4.41 ± 0.93 ^†^	0.76	0.61	0.70
Muscle mass in right leg (%)	12.35 ± 0.98 ^†^	12.61 ± 1.26 ^†^	0.23	0.79	0.92
**Pubertal**					
Weight (kg)	55.88 ± 12.94	52.09 ± 10.14	0.33		
Height (cm)	160.36 ± 7.90	161.83 ± 11.47	0.15		
Total BMI (kg/m^2^)	21.57 ± 3.97 *	19.77 ± 2.38	0.57		
Total body fat (%)	21.93 ± 6.62 *	17.88 ± 3.85	0.77		
Total muscle mass (%)	74.03 ± 6.26 *	77.85 ± 3.64	0.77		
Fat mass in left leg (%)	4.47 ± 1.29 *	3.72 ± 0.94	0.67		
Muscle mass in left leg (%)	12.66 ± 1.17 *	13.29 ± 1.30	0.51		
Fat mass in right leg (%)	4.45 ± 1.26 *	3.74 ± 0.98	0.63		
Muscle mass in right leg (%)	13.23 ± 1.23 *	13.86 ± 1.47	0.47		

*—The difference in means between cardiorespiratory fitness levels is significant at the 0.05 level. ^†^—The difference in means between age groups is significant at the 0.05 level. Abbreviations: BMI—body mass index; prepub—prepubertal group; pub—pubertal.

**Table 3:** Comparisons of respiratory variables, handgrip strength, and adherence to the Mediterranean diet.

	Cardiorespiratory Fitness		Effect Size		
	<P_75_	≥P_75_	<P_75_ vs. ≥P_75_	Prepub vs. Pub. <P_75_	Prepub vs. Pub. ≥P_75_
**Prepubertal**					
FVC (L)	2.82 ± 0.67 *^,†^	2.48 ± 0.54 ^†^	0.57	1.20	2.09
PEF (L/s)	4.59 ± 1.46 ^†^	4.27 ± 1.20 ^†^	0.24	0.44	1.09
FEV_1_ (L)	2.39 ± 0.62 ^†^	2.24 ± 0.50 ^†^	0.25	1.22	2.12
FEV_1_/FVC (%)	84.87 ± 9.11	86.48 ± 10.71	0.16	0.13	0.05
FEF_25–75_ (L/s)	2.74 ± 1.02 ^†^	2.64 ± 0.72 ^†^	0.11	0.93	1.58
Handgrip (kg)	23.21 ± 7.84 *^,†^	19.80 ± 6.13 ^†^	0.49	0.96	1.78
Handgrip (percentile)	69.79 ± 29.41 ^†^	60.91 ± 29.00	0.30	0.69	0.07
KIDMED index	6.91 ± 2.07	7.31 ± 1.97	0.20	0.02	0.16
**Pubertal**					
FVC (L)	3.66 ± 0.73 *	4.02 ± 0.93	0.43		
PEF (L/s)	5.13 ± 0.99	5.71 ± 1.45	0.48		
FEV_1_ (L)	3.14 ± 0.61 *	3.56 ± 0.74	0.63		
FEV_1_/FVC (%)	85.87 ± 5.88	86.87 ± 4.66	0.19		
FEF_25–75_ (L/s)	3.60 ± 0.84	4.01 ± 1.00	0.44		
Handgrip (kg)	30.98 ± 8.29	33.89 ± 9.70	0.32		
Handgrip (percentile)	50.27 ± 27.55	63.17 ± 31.28	0.44		
KIDMED index	6.86 ± 1.88	7.61 ± 1.75	0.41		

*—The difference in means between cardiorespiratory fitness levels is significant at the 0.05 level. ^†^—The difference in means between age groups is significant at the 0.05 level. Abbreviations: FVC—forced vital capacity; PEF—peak expiratory flow; FEV_1_—forced expiratory volume; FEF_25–75_, —mean forced expiratory flow between 25% and 75% of FVC; prepub—prepubertal group; pub—pubertal.

**Table 4:** Influence of body composition, respiratory variables handgrip strength, and adherence to the Mediterranean diet on cardiorespiratory fitness (stages of the 20-m shuttle run test (20 mSRT)).

	Coef.	Std. Err.	*p* > t	95% CI		
Age	0.34	0.08	*p* < 0.001	0.17	to	0.50
BMI (kg/m^2^)	−0.16	0.07	0.019	−0.29	to	−0.03
Total muscle mass (%)	0.11	0.04	0.003	0.04	to	0.18
FVC	0.57	0.24	0.017	0.10	to	1.04
Handgrip strength (kg)	0.01	0.02	0.628	−0.03	to	0.05
KIDMED index	0.06	0.05	0.271	−0.04	to	0.16
Constant	−4.85	3.30	0.143	−11.36	to	1.65
R^2^	0.52					

Abbreviations: BMI—body mass index; FVC—forced vital capacity; Std. Err.—Standard Error; CI—Confidence Interval.
